# The expression of V-ATPase is associated with drug resistance and pathology of non-small-cell lung cancer

**DOI:** 10.1186/1746-1596-8-145

**Published:** 2013-08-28

**Authors:** Qiang Lu, Sha Lu, Lijun Huang, Ting Wang, Yi Wan, Chang Xi Zhou, Cunhai Zhang, Zhipei Zhang, Xiaofei Li

**Affiliations:** 1 Department of Thoracic Surgery, Tangdu Hospital, Fourth Military Medical University, 710038 Xi’an, PR China; 2 Department of Intensive Care Unit, Xi’an Central Hospital, Xi’an, China; 3 Department of Vascular and Endocrine Surgery, Xijing Hospital, Fourth Military Medical University, Xi’an, China; 4 Department of Health Statistics & Institute for Health Informatics, Fourth Military Medical University, Xi’an, China; 5 Department of Nanlou Respiratory Diseases, PLA General Hospital, Beijing, China; 6 Department of Intensive Care Unit, 117th Hospital of PLA, Hangzhou, China

**Keywords:** Non-small cell lung cancer, V-ATPase, Drug resistance, Drug sensitivity

## Abstract

**Objective:**

This article aims to investigate the expression of vacuolar-H + −ATPase (V-ATPase) in non-small cell lung cancer (NSCLC) and its variations with pathological type and grade. Furthermore, to evaluate the chemotherapy drug sensitivity of different cancer tissues as well as its correlation with V-ATPase expression in NSCLC.

**Methods:**

V-ATPase expression was examined in 92 NSCLC tissue samples using the immunohistochemical Envision method and immunofluorescence assay. The location of V-ATPase expression was observed by confocal laser scanning microscopy and the difference of its expression rate was evaluated. The sensitivity of cancer tissues to chemotherapy drug was examined using MTT assay and its correlation with the V-ATPase expression was tested in NSCLC by Spearman rank correlation analysis.

**Results:**

V-ATPase expression was mainly localized in the cell membrane and cytoplasm. The expression rate of V-ATPase was 71.43% in squamous cell lung cancer, significantly lower than that of the lung adenocarcinoma (83.72%, *P* = 0.000). In different pathological grades of squamous cell lung cancer, the expression rate of V-ATPase was 58.33% in grade II, significantly lower than that of the grade III (84.00%, *P* = 0.014). The expression rate of V-ATPase in grade II lung adenocarcinoma was 76.67%, significantly lower than that of the grade ΙΙΙ adenocarcinoma (100.0%, *P* = 0.012). Correlation analysis showed that the sensitivity of NSCLC tissues to cyclophosphamide, gemcitabine, doxorubicin, paclitaxel and cisplatin was significantly correlated with the V-ATPase expression rate (*P* < 0.05).

**Conclusions:**

V-ATPase was overexpressed in NSCLC. The expression of V-ATPase was related to the pathological type and grade of cancer and was likely associated with chemotherapy drug resistance in NSCLC.

**Virtual slides:**

The virtual slide(s) for this article can be found here: http://www.diagnosticpathology.diagnomx.eu/vs/7515811511020000

## Introduction

The morbidity and mortality of lung cancer have increased over the years. Lung cancer has become the leading malignancy in patients, causing great threats to human health. At present, lung cancer is commonly treated with multidisciplinary treatment strategies, of which chemotherapy is the primary approach. However, chemotherapy drug resistance is an important cause of treatment failure in lung cancer [1-3]. Researchers showed that an acidic microenvironment can significantly affect the malignant behaviors of cancer cells related to invasion, metastasis and drug resistance [4,5]. The drug resistance of cancer cells is likely to be related to the changes in pH gradient between the extracellular environment and the cytoplasm. Vacuolar-H + −ATPase (V-ATPase) plays a major role in the regulation of cellular pH conditions [6,7].

Previous studies focused on V-ATPase were mainly conducted on cancer metastasis and invasion aspects [8-11]. However, there are no detailed reports on the correlation between V-ATPase expression and drug resistance in tumor cells. In this study, we examined the expression of V-ATPase in different pathological types of non-small cell lung cancer (NSCLC) by immunohistochemical and immunofluorescence assays, followed by the evaluation of the drug resistance in NSCLC tissues. The results were used to explore the correlation between V-ATPase expression and chemotherapy drug resistance in NSCLC. This work presents the first evidence of high V-ATPase expression in NSCLC. The expression of V-ATPase was shown to be related to the pathological type and grade of the cancer and might be associated with the chemotherapy drug resistance in NSCLC.

## Materials and methods

During May 2011 to April 2012, clinical specimens were collected from 92 patients of primary NSCLC who underwent thoracic surgery in Tangdu Hospital, Fourth Military Medical University. The patients included 66 males and 26 female with an average age of 63.2 ± 13.1. All patients were classified according to the lung cancer classification and the evaluation criteria were jointly announced by the International Society for the Study of Lung Cancer, the American Thoracic Society and the European Respiratory Society. The experiments were conducted with NSCLC specimens of two histological types of lung cancer including squamous cell lung cancer and adenocarcinoma (Table 1).

**Table 1:** Patient characteristics (NSCLC,n = 92)

**Variable**	**Result**
Age, n (%)	
≤ 60	43(46.74)
> 60	49(53.26)
Sex, n (%)	
Male	66(71.74)
Female	26(28.26)
TNM stages, n (%)	
II	54(58.70)
III	38(41.30)
Cell types, n (%)	
Squamous cell lung cancer	49(71.42)
Lung adenocarcinoma	43(83.72)

All the 45 smoking patients were male and pack-years of them were fifteen in average. There were 12 female patients suffered passive smoking. 23 female patients had the history of exposure to cooking fume and one to asbestos. 22 people have exposure history of diesel exclusion, which were include 17 male patients and 5 female patients.

Immediately after the clinical specimens were collected in the surgery, 1.5–2.0 cm^3^ specimens were cut and placed in sterile tubes pre-filled with sterile RPMI 1640 culture medium. Part of the fresh cancer tissue was used for testing the susceptibility to chemotherapy drugs. The specimens were fixed with 10% formalin and were embedded in paraffin after routine dehydration and transparency. Histopathological typing and grading were performed by the Department of Pathology and clinical typing after the surgery. All patients agreed to sign written informed consents. The study was approved by the Ethics Committee of the hospital.

### Reagents

The V-ATPase protein antibody (commercialized mouse anti-human, Molecular Probes) was purchased from Jingmei (Shenzhen, China). An Envision™ kit for immunohistochemical analysis (Dako) and the secondary antibody for immunofluorescence assay were purchased from Santa Crus Biotechnoloty (Shanghai, China).

Chemotherapy drugs including: cyclophosphamide, gemcitabine, doxorubicin, paclitaxel and cisplatin were purchased from Jiangsu Ruiheng Co., Ltd (Lianyungang, China), Lilly France (Fegersheim, France), Zhejiang Hisun Pharmaceutical Corporation (Zhejiang, China), Bristol-Myers Squibb Co. (Tokyo, Japan) and Shandong Qilu Pharmaceutical Co., Ltd (Jinan, China), respectively. The RPMI1640 media was purchased from Gibco Co (CA, USA). Fetal bovine serum was purchased from Zhejiang Tianhang Biological Technology Co., Ltd (Hangzhou, China). Trypsin was purchased from Hyclone Co. (Logan, UT, USA.). The MTT and DMSO were purchased from Sigma-Aldrich (St. Louis, MO, USA).

### Cell suspension preparation and drug sensitivity test

Fresh cancer tissue was repeatedly washed with saline solution containing penicillin-streptomycin. After the fat and necrotic tissues were removed, the cell suspension was prepared with the cancer tissue and was filtered through a 200-mesh sieve of steel-net. The cell density in the suspension was adjusted to 5 × 10^5^ to 10^7^/ml with the 1640 complete culture medium and the cells were counted by trypan blue staining. The diluted cell suspension was inoculated to 96-well culture plates (190 μl/well) in four groups including: blank, control and experimental groups of high- and low-level drug groups (four wells per group). The inoculated plates were incubated at 37°C in a 5%-CO_2_ incubator for 24 h. In the next step, 10-μl aliquots of the abovementioned chemotherapy drugs were added to the high-level drug group with final concentrations adjusted to the human plasma concentration (12, 2, 0.4, 0.5 and 1 mg/ml, respectively). The drugs used in the low-level drug group were correspondingly diluted four times.

### Chemosensitivity assay

After 48-h incubation, 20 μl of MTT reagent (5 mg/ml) was added to each well. The plates were incubated for 4 h. The culture supernatant was removed and 150 μl of DMSO was added to each well. The plates were oscillated for 5 min to dissolve MTT formazan crystals. The absorbance of each well was determined at 570 nm using a microplate reader.

### Calculation of the drug sensitivity

#### Calculation of drug inhibition rate (IR)

The drug inhibition and cell survival rates were calculated based on the following formula:

Drug inhibition rate (%) = (Optical density of control group-optical density of drug group)/(optical density of control group-optical density of blank group) × 100%

Cell survival rate = Drug group/Control group

#### Criteria for chemotherapy drug sensitivity

The criteria for chemotherapy drug sensitivity as followings: the IR of less than 30% was considered as drug resistance; 30–50% as low sensitivity; higher than 50% as medium sensitivity and higher than 70% as high sensitivity.

### Immunohistochemical assay of V-ATPase protein

The specimens were prepared as 5-μm serially sectioned slides and were used for HE and immunohistochemical staining. The immunohistochemical staining included two steps (Envision™). First, the slices were dewaxed to water, digested with urea and blocked with 3% hydrogen peroxide. After the microwave repair with a citric acid buffer, the slides were cooled, blocked with 10% goat serum and incubated with the primary antibody at 4°C overnight. Second, the slides were taken from the refrigerator and were recovered to 37°C. The anti-rabbit secondary antibody (EnVision) was added for reaction at 37°C temperature. The slides were washed with phosphate buffer saline (PBS) between each intermediate step. After the diaminobenzidine staining, the slides were examined by microscopy. The staining was terminated and the slides were slightly stained with hematoxylin. After dehydration and transparency treatments, the slides were examined by light microscopy. The control group was prepared with positive, negative and blank controls.

### Immunofluorescence assay of V-ATPase protein

The specimens were incubated with the primary antibody following the same procedure as described above. The slides were recovered to 37°C on the following day and TRITC-labeled red fluorescent anti-mouse antibody was added. The slides were incubated at 37°C for 40 min, washed with PBS, mounted with glycerin, examined by laser confocal microscopy and photographed.

### Scoring of immunohistochemical data

The V-ATPase protein expression was mainly localized in the cytoplasm and nuclear membrane. According to the semi-quantitative integration method, five random fields of view were observed for each specimen at high magnification (×400). The results were scored based on the following criteria: First, positive cells (≤5%: 0; 6–25%: 1; 26–50%: 2; 51–75%: 3 and >75%: 4). Second, positive intensity (yellow: 1; brown: 2 and tan: 3). Third, cell positive rate integrally multiplied by the staining intensity: [(0: negative (−), 1–4: weakly positive (+); 5–8: moderately positive (++) and 9–12: strongly positive (+++)] [11].

### Statistical methods

Statistical analysis was performed with SPSS software. Data comparison among squamous cell carcinoma and adenocarcinoma, pathological grades of squamous cell carcinoma, and pathological grades of adenocarcinoma were done using the Mann–Whitney U rank sum test. The correlation between the drug sensitivity of cancer tissues and the expression of V-ATPase was analyzed by Spearman rank correlation analysis. The rank correlation coefficients were expressed as rs (*P* < 0.05, rs < −0.3 was considered as negatively correlated).

## Results

### Immunohistochemistry and immunofluorescence data

**Figure 1:**
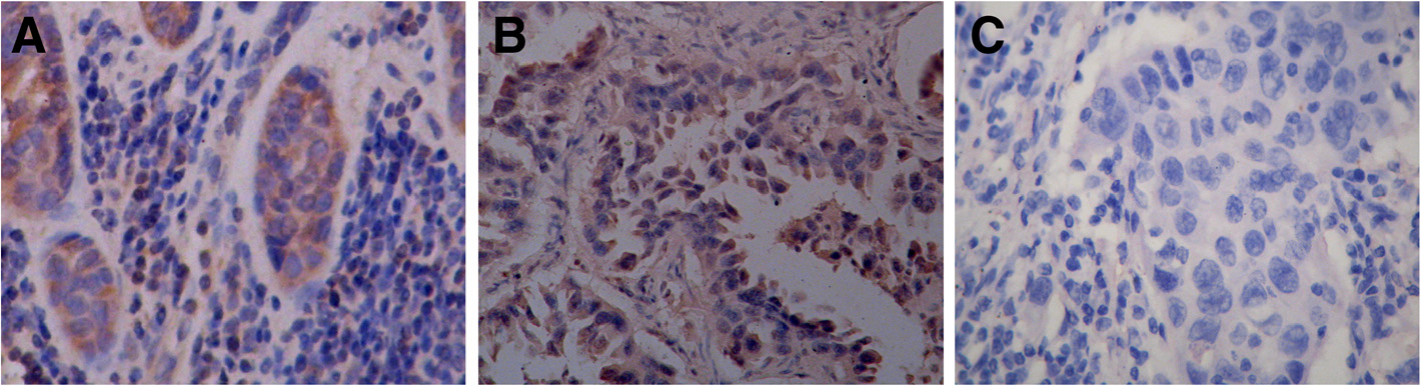
**Expression of V-ATPase in NSCLC. (A)**: squamous cell lung cancer; **(B)**: lung adenocarcinoma; **(A,B)**: positive staining of V-ATPase in the plasma membrane and cytoplasm; **(C)**: blank (EnVision; 400×).

**Figure 2:**
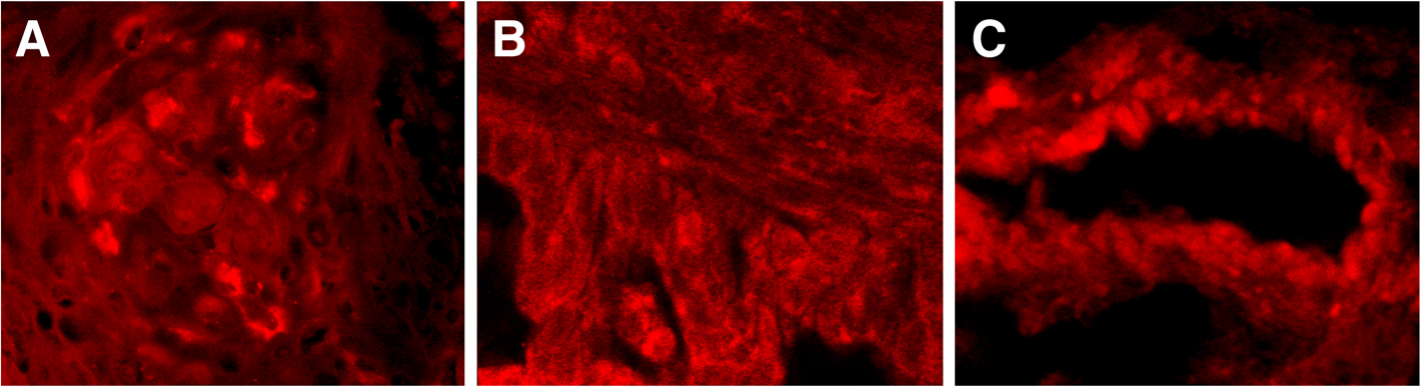
**Location of V-ATPase in NSCLC. (A)**: squamous cell lung cancer; **(B)** and **(C)**: lung adenocarcinoma; positive staining of V-ATPase in the plasma membrane and cytoplasm (1000×).

The results of the immunohistochemical assay showed that the V-ATPase expression was mainly localized in the cell membrane and cytoplasm (Figure 1), whereas the immunofluorescence staining was mainly localized in the cell membrane (Figure 2). The total expression rate of V-ATPase in squamous cell lung cancer was 71.43% (Table 2), significantly lower than that of the lung adenocarcinoma (83.72%, *P* = 0.000). Among different pathological grades of squamous cell lung cancer, the expression rate of V-ATPase was 58.33% in grade II, significantly lower than that of the grade III (84.00%, 0.014). As for the adenocarcinoma, the expression rate of V-ATPase was 76.7% in grade II adenocarcinoma (Table 2), significantly lower than that of the grade III adenocarcinoma (100.0%, *P* = 0.012).

**Table 2:** Expression of V-ATPase in different differentiations of NSCLC

**Typing**	**n**	**Pathological grading**	**V-ATPvase positive intensity**			**Positive rate %**	**p-value**	
			**-**	**+**	**++**			
Squamous cell lung cancer	49	II	10	14	0	58.33	U = 195.0	U = 593.5
		III	4	17	4	84	p = 0.014	
Lung adenocarcinoma	43	II	7	11	12	76.67	U = 101.0	p = 0.000
		III	0	2	11	100	p = 0.000	

### Correlation between cancer tissue chemotherapy drug sensitivity and associated V-ATPase expression

The sensitivity of the lung cancer tissues to cyclophosphamide, gemcitabine, doxorubicin, paclitaxel, and cisplatin and the expression of V-ATPase in NSCLC tissues had a *P* value of less than 0.05. The corresponding correlation coefficients (rs) were -0.697, -0.654, -0.598, -0.216 and -0.604, respectively (Table 3). Similarly, the *P*-values of the correlation test was less than 0.05 (Table 4) for cyclophosphamide, gemcitabine, doxorubicin, paclitaxel, and cisplatin in squamous cell lung cancer (rs = -0.584, -0.512, -0.544, -0.269 and −0.306, respectively). Furthermore, the *P*-values of the correlation test was less than 0.05 (Table 5) for cyclophosphamide, gemcitabine, doxorubicin and cisplatin (rs = -0.742, -0.607, -0.63, -0.349 and −0.707).

**Table 3:** The correlation of V-ATPase expression with drug sensitivity in NSCLC

**V-ATPase**	**Cyclophosphamide**					**Gemcitabine**					**Doxorubicin**					**Paclitaxel**					**Cisplatin**				
	**R**	**LS**	**MS**	**HS**	**p value**	**R**	**LS**	**MS**	**HS**	**p value**	**R**	**LS**	**MS**	**HS**	**p value**	**R**	**LS**	**MS**	**HS**	**p value**	**R**	**LS**	**MS**	**HS**	**p value**
_	0	12	6	3	χ^2^ = 53.3	0	4	11	6	χ^2^ = 48.13	0	3	6	12	χ^2^ = 41.09	1	0	2	18	χ^2^ = 22.35	2	2	8	9	χ^2^ = 46.9
+	14	26	4	0	p = 0.000	6	12	24	2	p = 0.000	16	10	12	6	p = 0.000	1	1	10	32	p = 0.001	6	13	17	8	p = 0.000
++	24	3	0	0	rs = −0.697	14	13	0	0	rs = −0.654	15	11	1	0	rs = −0.598	6	3	7	11	rs = −0.216	21	4	1	1	rs = −0.604

**Table 4:** The correlation of V-ATPase expression with drug sensitivity (resistance) in squamous cell lung cancer

**V-ATPase**	**Cyclophosphamide**					**Gemcitabine**					**Doxorubicin**					**Paclitaxel**					**Cisplatin**				
	**R**	**LS**	**MS**	**HS**	**p value**	**R**	**LS**	**MS**	**HS**	**p value**	**R**	**LS**	**MS**	**HS**	**p value**	**R**	**LS**	**MS**	**HS**	**p value**	**R**	**LS**	**MS**	**HS**	**p value**
_	0	3	2	2	χ2 =30.12	0	2	3	2	χ2 = 24.143	0	2	0	5	χ2 = 34.11	0	0	0	0	χ2 = 10.03	0	0	2	5	χ2 = 32.88
+	4	7	2	0	p = 0.00	3	6	4	0	p = 0.000	0	4	5	1	p = 0.000	0	1	5	7	p = 0.124	3	3	6	1	p = 0.000
++	20	3	0	0	rs = −0.742	12	11	0	0	rs = −0.607	0	9	1	0	rs = −0.630	4	3	5	11	rs = −0.349	17	4	1	1	rs = −0.707

*R*: Resistance; *LS*: Low sensitivity; *MS*: Moderate sensitivity; *HS*: High sensitivity.

**Table 5:** The correlation of V-ATPase expression with drug sensitivity (resistance) in lung adenocarcinoma

**V-ATPase**	**Cyclophosphamide**					**Gemcitabine**					**Doxorubicin**					**Paclitaxel**					**Cisplatin**				
	**R**	**LS**	**MS**	**HS**	**p value**	**R**	**LS**	**MS**	**HS**	**p value**	**R**	**LS**	**MS**	**HS**	**p value**	**R**	**LS**	**MS**	**HS**	**p value**	**R**	**LS**	**MS**	**HS**	**p value**
_	0	9	4	1	χ^2^ = 20.68	0	2	8	4	χ^2^ = 21.45	0	1	6	7	χ^2^ = 17.47	1	0	2	11	χ^2^ = 15.12	2	2	6	4	χ^2^ = 21.04
+	10	19	2	0	p = 0.002	3	6	20	2	p = 0.002	13	6	7	5	p = 0.008	1	0	5	25	p = 0.004	5	10	11	7	p = 0.002
++	4	0	0	0	rs = −0.584	2	2	0	0	rs = −0.512	2	2	0	0	rs = −0.544	2	0	2	0	rs = −0.269	4	0	0	0	rs = −0.306

*R*: Resistance; *LS*: Low sensitivity; *MS*: Moderate sensitivity; *HS*: High sensitivity.

## Discussion

Although antineoplastic agents have an important role in the treatment of NSCLC, their treatment efficiency is commonly low due to the tumor cell resistance. The membrane transport proteins play a key role in drug metabolism. In particular, the drug efflux process mediated by ATP-binding cassette (ABC) transporter plays a major role in the chemotherapy drug resistance in cancer cells [7]. A large body of work has been conducted on the drug resistance-related Multi-drug resistance 1 (MDR1), multidrug resistance-associated protein (MRP) and ATP-binding cassette transporter 2 (ABCG 2), which function through different mechanisms [12-16].

Molecular mechanisms of tumor cell resistance were another hot point. It was found that molecular pathological pathways of lung cancer were related with potential drug influence also. Structural of EGFR changes leading to the activating properties. Insertions in exon 19 are likely to respond to TKI therapy [17]. Lung adenocarcinoma with predominant SMPC (stromal invasive micropapillary component) may be associated with a poor prognosis and have different phenotypic and genotypic characteristics [18].

ATPase ion pump is an ATP-dependent active transport carrier, which transports Na^+^, K^+^, H^+^, Ca^2+^ and Cu^2+^ out of the cells and organelles. V-ATPase is a macromolecular complex enzyme of ATPase and is expressed on the vacuolar membrane of cytoplasm (microsome), as well as the cell membrane. V-ATPase produces or maintains the transmembrane electrochemical ionic gradient that is related to the accumulation, intracellular distribution and sensitivity of the anticancer drugs [19]. The overexpression of V-ATPase in tumor cells is of great significance to the maintenance of cytoplasmic alkaline environment, promotion of tumor cell growth, improvement of the extracellular acid environment, promotion of cell invasion and metastasis [8,20]. Furthermore, it induces the invasive phenotype in the tumor cells [21,22]. The transport capacity of ABCG2 for methotrexate, folic acid, mitoxantrone and topotecan can be enhanced under low pH conditions in tumor cell lines, such that at pH 5.5, the transport capacity of ABCG2 for drugs is five times higher than that of the normal conditions [23,24]. The drug resistance could be possibly related to the changes in the pH gradient between the extracellular environment and the cytoplasm. It is believed that V-ATPase plays a major role in the regulation of intracellular pH value [25-27]. Available data on V-ATPase have mainly been reported based on the plant studies [28,29] with the exception of a few reports on liver, breast, pancreatic, esophageal, gastric carcinomas as well as melanoma cells [8-11]. However, no reports are available on the correlation between the V-ATPase expression in NSCLC and the drug resistance in the relevant cancer tissues.

In this study, the results of the immunohistochemical and immunofluorescence assays showed that V-ATPase was overexpressed in NSCLC, while its expression rate was significantly lower in the adjacent normal tissue (data not shown). In terms of different histological types of NSCLC, the V-ATPase expression rate was 71.43% and 83.72% in squamous cell lung cancer and lung adenocarcinoma. The Mann–Whitney rank sum test indicated that there were significant differences in the V-ATPase expression rate between the two cancer tissues (*P* < 0.001). The rank sum test among different grades of squamous cell lung cancer and different differentiation degrees of adenocarcinoma further showed significant differences (*P* = 0.014 and 0.012, respectively). These results suggested that was overexpressed in NSCLC. Furthermore, it was demonstrated that the V-ATPase expression rate of lung adenocarcinoma was higher than that of the squamous cell lung cancer and higher in grade ΙΙΙ adenocarcinoma than that of the well-differentiated adenocarcinoma. In clinical practice, it is commonly found that lung adenocarcinoma is more resistant to chemotherapy drugs than squamous cell lung cancer, and that the resistance level is often related to the degree of malignancy. Together these findings indicated that the V-ATPase expression could be potentially related to the drug resistance in NSCLC.

In order to further examine the relationship between the V-ATPase expression and the drug resistance in NSCLC, we carried out the cancer tissue drug sensitivity test. Spearman rank correlation analysis was performed for the results of the drug sensitivity test of cyclophosphamide, gemcitabine, doxorubicin, paclitaxel and cisplatin in NSCLC tissues and for the V-ATPase expression in the corresponding tissues. All the obtained *P*-values were less than 0.05 and all the coefficients of rank correlation were less than −0.30. The correlation was found to be higher in lung adenocarcinoma as compared to that of the squamous cell lung cancer. However, this correlation was not significant for paclitaxel when tested separately in squamous cell lung cancer and lung adenocarcinoma. Overall, there was a negative correlation between the V-ATPase expression and drug sensitivity in the NSCLC tissues. Therefore, the V-ATPase expression had a strong positive correlation with the drug resistance in the NSCLC tissues. This finding suggested that the V-ATPase expression could be related to the drug resistance in the NSCLC tissues. The V-ATPase expression rate had a stronger correlation with the drug resistance in the lung adenocarcinoma tissue as compared to that of the squamous cell lung cancer tissue.

Although this study did not prove that the expression of V-ATPase was directly linked to the drug resistance of cancer tissues, other experiments have already shown that the activity of V-ATPase was increased in multidrug-resistant cell lines. Its subunit genes were upregulated under the action of antineoplastic agents [30,31]. Therefore, we speculated that V-ATPase was related to the drug resistance in tumor tissues. However, further experiments are needed to unravel the exact mechanism.

## Competing interest

All of the authors in this manuscript declared that no financial interests were related to this study or future application after publication.

## Authors’ contributions

All authors contributed to this work. QL participated in the design of the study. SL and LH conceived of the study, participated in its design. TW helped draft the manuscript. YW performed the statistical analysis.CXZ and CZ helped search articles. ZZ was involved in the direct clinical care (diagnosis, decision making, and treatment) of the reported patient. XL revised the draft. All authors read and approved the final manuscript.

## References

[B1] Owonikoko TK Behera M Chen Z Bhimani C Curran WJ Khuri FR Ramalingam SS A systematic analysis of efficacy of second-line chemotherapy in sensitive and refractory small-cell lung cancer J Thorac Oncol 2012 7 866 872 10.1097/JTO.0b013e31824c7f4b 22722788 PMC3381878

[B2] Ho CC Kuo SH Huang PH Huang HY Yang CH Yang PC Caveolin-1 expression is significantly associated with drug resistance and poor prognosis in advanced non-small cell lung cancer patients treated with gemcitabine-based chemotherapy Lung Cancer 2008 59 105 110 10.1016/j.lungcan.2007.07.024 17850918

[B3] Ohashi K Sequist LV Arcila ME Moran T Chmielecki J Lin YL Pan Y Wang L de Stanchina E Shien K Lung cancers with acquired resistance to EGFR inhibitors occasionally harbor BRAF gene mutations but lack mutations in KRAS, NRAS, or MEK1 Proc Natl Acad Sci U S A 2012 109 E2127 E2133 10.1073/pnas.1203530109 22773810 PMC3411967

[B4] Peebles KA Lee JM Mao JT Hazra S Reckamp KL Krysan K Dohadwala M Heinrich EL Walser TC Cui X Inflammation and lung carcinogenesis: applying findings in prevention and treatment Expert Rev Anticancer Ther 2007 7 1405 1421 10.1586/14737140.7.10.1405 17944566

[B5] Huang Y Sadee W Membrane transporters and channels in chemoresistance and -sensitivity of tumor cells Cancer Lett 2006 239 168 182 10.1016/j.canlet.2005.07.032 16169662

[B6] Sasazawa Y Futamura Y Tashiro E Imoto M Vacuolar H + −ATPase inhibitors overcome Bcl-xL-mediated chemoresistance through restoration of a caspase-independent apoptotic pathway Cancer Sci 2009 100 1460 1467 10.1111/j.1349-7006.2009.01194.x 19459857 PMC11159986

[B7] Nishisho T Hata K Nakanishi M Morita Y Sun-Wada GH Wada Y Yasui N Yoneda T The a3 isoform vacuolar type H(+)-ATPase promotes distant metastasis in the mouse B16 melanoma cells Mol Cancer Res 2011 9 845 855 10.1158/1541-7786.MCR-10-0449 21669964

[B8] Hendrix A Sormunen R Westbroek W Lambein K Denys H Sys G Braems G Van den Broecke R Cocquyt V Gespach C V-ATPase expression and activity is required for Rab27B-dependent invasive growth and metastasis of breast cancer Int J Cancer 2013 133 4 843 845 10.1002/ijc.28079 23390068

[B9] Hinton A Sennoune SR Bond S Fang M Reuveni M Sahagian GG Jay D Martinez-Zaguilan R Forgac M Function of a subunit isoforms of the V-ATPase in pH homeostasis and in vitro invasion of MDA-MB231 human breast cancer cells J Biol Chem 2009 284 16400 16408 10.1074/jbc.M901201200 19366680 PMC2713521

[B10] Straud S Zubovych I De Brabander JK Roth MG Inhibition of iron uptake is responsible for differential sensitivity to V-ATPase inhibitors in several cancer cell lines PLoS One 2010 5 e11629 10.1371/journal.pone.0011629 20661293 PMC2905441

[B11] Huang L Lu Q Han Y Li Z Zhang Z Li X ABCG2/V-ATPase was associated with the drug resistance and tumor metastasis of esophageal squamous cancer cells Diagn Pathol 2012 7 180 10.1186/1746-1596-7-180 23244569 PMC3542252

[B12] Campa D Muller P Edler L Knoefel L Barale R Heussel CP Thomas M Canzian F Risch A A comprehensive study of polymorphisms in ABCB1, ABCC2 and ABCG2 and lung cancer chemotherapy response and prognosis Int J Cancer 2012 131 2920 2928 10.1002/ijc.27567 22473764

[B13] Yamazaki R Nishiyama Y Furuta T Hatano H Igarashi Y Asakawa N Kodaira H Takahashi H Aiyama R Matsuzaki T Novel acrylonitrile derivatives, YHO-13177 and YHO-13351, reverse BCRP/ABCG2-mediated drug resistance in vitro and in vivo Mol Cancer Ther 2011 10 1252 1263 10.1158/1535-7163.MCT-10-0874 21566063

[B14] Albermann N Schmitz-Winnenthal FH Z'Graggen K Volk C Hoffmann MM Haefeli WE Weiss J Expression of the drug transporters MDR1/ABCB1, MRP1/ABCC1, MRP2/ABCC2, BCRP/ABCG2, and PXR in peripheral blood mononuclear cells and their relationship with the expression in intestine and liver Biochem Pharmacol 2005 70 949 958 10.1016/j.bcp.2005.06.018 16054595

[B15] Modok S Mellor HR Callaghan R Modulation of multidrug resistance efflux pump activity to overcome chemoresistance in cancer Curr Opin Pharmacol 2006 6 350 354 10.1016/j.coph.2006.01.009 16690355

[B16] Nakagawa H Saito H Ikegami Y Aida-Hyugaji S Sawada S Ishikawa T Molecular modeling of new camptothecin analogues to circumvent ABCG2-mediated drug resistance in cancer Cancer Lett 2006 234 81 89 10.1016/j.canlet.2005.05.052 16309825

[B17] Otto C Csanadi A Fisch P Werner M Kayser G Molecular modeling and description of a newly characterized activating mutation of the EGFR gene in non-small cell lung cancer Diagn Pathol 2012 7 146 10.1186/1746-1596-7-146 23088930 PMC3523061

[B18] Ohe M Yokose T Sakuma Y Miyagi Y Okamoto N Osanai S Hasegawa C Nakayama H Kameda Y Yamada K Isobe T Stromal micropapillary component as a novel unfavorable prognostic factor of lung adenocarcinoma Diagn Pathol 2012 7 3 10.1186/1746-1596-7-3 22225786 PMC3320518

[B19] Perez-Sayans M Garcia-Garcia A Scozzafava A Supuran CT Inhibition of V-ATPase and carbonic anhydrases as interference strategy with tumor acidification processes Curr Pharm Des 2012 18 1407 1413 10.2174/138161212799504876 22360559

[B20] Wiedmann RM von Schwarzenberg K Palamidessi A Schreiner L Kubisch R Liebl J Schempp C Trauner D Vereb G Zahler S The V-ATPase-inhibitor archazolid abrogates tumor metastasis via inhibition of endocytic activation of the Rho-GTPase Rac1 Cancer Res 2012 72 5976 5987 10.1158/0008-5472.CAN-12-1772 22986742

[B21] Sennoune SR Bakunts K Martinez GM Chua-Tuan JL Kebir Y Attaya MN Martinez-Zaguilan R Vacuolar H + −ATPase in human breast cancer cells with distinct metastatic potential: distribution and functional activity Am J Physiol Cell Physiol 2004 286 C1443 C1452 10.1152/ajpcell.00407.2003 14761893

[B22] Niikura K Effect of a V-ATPase inhibitor, FR202126, in syngeneic mouse model of experimental bone metastasis Cancer Chemother Pharmacol 2007 60 555 562 10.1007/s00280-006-0401-8 17187252

[B23] Breedveld P Pluim D Cipriani G Dahlhaus F van Eijndhoven MA de Wolf CJ Kuil A Beijnen JH Scheffer GL Jansen G The effect of low pH on breast cancer resistance protein (ABCG2)-mediated transport of methotrexate, 7-hydroxymethotrexate, methotrexate diglutamate, folic acid, mitoxantrone, topotecan, and resveratrol in in vitro drug transport models Mol Pharmacol 2007 71 240 249 17032904 10.1124/mol.106.028167

[B24] Li L Sham YY Bikadi Z Elmquist WF pH-Dependent transport of pemetrexed by breast cancer resistance protein Drug Metab Dispos 2011 39 1478 1485 10.1124/dmd.111.039370 21628496

[B25] Mahoney BP Raghunand N Baggett B Gillies RJ Tumor acidity, ion trapping and chemotherapeutics. I. Acid pH affects the distribution of chemotherapeutic agents in vitro Biochem Pharmacol 2003 66 1207 1218 10.1016/S0006-2952(03)00467-2 14505800

[B26] Pastor-Soler N Beaulieu V Litvin TN Da Silva N Chen Y Brown D Buck J Levin LR Breton S Bicarbonate-regulated adenylyl cyclase (sAC) is a sensor that regulates pH-dependent V-ATPase recycling J Biol Chem 2003 278 49523 49529 10.1074/jbc.M309543200 14512417 PMC3652382

[B27] Recchi C Chavrier P V-ATPase: a potential pH sensor Nat Cell Biol 2006 8 107 109 10.1038/ncb0206-107 16450005

[B28] Kim HS Oh JM Luan S Carlson JE Ahn SJ Cold stress causes rapid but differential changes in properties of plasma membrane H(+)-ATPase of camelina and rapeseed J Plant Physiol 2013 170 9 828 837 10.1016/j.jplph.2013.01.007 23399403

[B29] Tang RJ Liu H Yang Y Yang L Gao XS Garcia VJ Luan S Zhang HX Tonoplast calcium sensors CBL2 and CBL3 control plant growth and ion homeostasis through regulating V-ATPase activity in Arabidopsis Cell Res 2012 22 1650 1665 10.1038/cr.2012.161 23184060 PMC3515760

[B30] Torigoe T Izumi H Ishiguchi H Uramoto H Murakami T Ise T Yoshida Y Tanabe M Nomoto M Itoh H Kohno K Enhanced expression of the human vacuolar H + −ATPase c subunit gene (ATP6L) in response to anticancer agents J Biol Chem 2002 277 36534 36543 10.1074/jbc.M202605200 12133827

[B31] Nasiri N Shokri E Nematzadeh GA Aeluropus littoralis NaCl-induced vacuolar H(+)-ATPase subunit c: molecular cloning and expression analysis Genetika 2012 48 1380 1388 23516899

